# Lipid Profile and Cardiovascular Risk Modification after Hepatitis C Virus Eradication

**DOI:** 10.3390/pathogens13040278

**Published:** 2024-03-25

**Authors:** Andrea Pascual-Oliver, Diego Casas-Deza, Carmen Yagüe-Caballero, Jose M. Arbones-Mainar, Vanesa Bernal-Monterde

**Affiliations:** 1 Gastroenterology Department, Miguel Servet University Hospital, 50009 Zaragoza, Spain; apascualoliver@gmail.com (A.P.-O.); carmenyaguecaballero@gmail.com (C.Y.-C.); vbernalm@gmail.com (V.B.-M.); 2 Adipocyte and Fat Biology Laboratory (AdipoFat), Translational Research Unit, University Hospital Miguel Servet, 50009 Zaragoza, Spain; jmarbones.iacs@aragon.es; 3 Instituto Aragones de Ciencias de la Salud (IACS), 50009 Zaragoza, Spain; 4 Instituto de Investigación Sanitaria (IIS) Aragon, 50009 Zaragoza, Spain; 5 CIBER Fisiopatología Obesidad y Nutrición (CIBERObn), Instituto Salud Carlos III, 28029 Madrid, Spain

**Keywords:** hepatitis C virus, lipoproteins, cardiovascular disease, direct-acting antiviral treatment, chronic hepatitis C virus treatment, insulin resistance

## Abstract

The eradication of the hepatitis C virus (HCV) has revolutionized the hepatology paradigm, halting the progression of advanced liver disease in patients with chronic infection and reducing the risk of hepatocarcinoma. In addition, treatment with direct-acting antivirals can reverse the lipid and carbohydrate abnormalities described in HCV patients. Although HCV eradication may reduce the overall risk of vascular events, it is uncertain whether altered lipid profiles increase the risk of cerebrovascular disease in certain patients. We have conducted a review on HCV and lipid and carbohydrate metabolism, as well as new scientific advances, following the advent of direct-acting antivirals.

## 1. Introduction

The hepatitis C virus (HCV) is recognized as a significant human pathogen that initially causes acute hepatitis. However, it has the potential to evolve into chronic hepatitis, leading to severe liver complications such as cirrhosis and hepatocellular carcinoma, posing a substantial global public health challenge. As a single-stranded RNA virus belonging to the Flaviviridae family, HCV’s mechanism of infection and replication is complex. It involves evading the host’s immune response, contributing to its chronicity in infected individuals. The virus’s genetic diversity, marked by multiple genotypes and subtypes, complicates vaccine development and treatment strategies. The progression from acute to chronic HCV infection stresses the importance of early detection and effective antiviral therapies to prevent long-term liver damage and reduce the risk of liver cancer. Despite advances in treatment, HCV remains a leading cause of liver transplantation worldwide, highlighting the need for continued research and public health efforts to combat this virus [1].

HCV transmission occurs primarily through blood-to-blood contact. In healthcare environments, reusing or inadequately sterilizing medical equipment, notably syringes and needles, presents a significant risk. Additionally, the transfusion of blood and blood products that have not undergone thorough screening processes can serve as a conduit for HCV transmission. Another prevalent route is through the sharing of injection equipment among individuals using injectable drugs [2].

HCV is classified into seven genotypes, with multiple subtypes, which are unevenly distributed geographically and differ in response to treatment [3].

## 2. Epidemiology of HCV Infection and Clinical Course

The prevalence of HCV infection has been declining since the second half of the 20th century [4]. This is due to improved hygienic and dietary conditions in developing countries and active surveillance in high-incidence countries. Together, these strategies have played a pivotal role in reducing the global burden of HCV, showcasing the importance of comprehensive public health initiatives in combating infectious diseases.

However, accurate estimates of global HCV prevalence are difficult to establish due to underdiagnosis, underreporting, and a lack of routine surveillance in most countries [5]. The estimated global prevalence of HCV viremia in early 2020 was 0.7 percent, reflecting 56.8 million people with chronic HCV infection. These data reflect a decrease in prevalence compared to 2015 when there were 63.6 million chronic HCV infections, representing 0.9 percent of the global population.

In Europe, the main incidence areas are the Eastern Mediterranean countries (62.5 per 100,000), where it is associated with healthcare, and the Eastern European region (61.8 per 100,000), where it is associated with injectable drug use [6].

Hepatitis C often progresses stealthily, mirroring other liver diseases with an initial asymptomatic phase in most cases. Over time, it may lead to cirrhosis, presenting complications such as ascites, variceal bleeding, and hepatic encephalopathy. A notable distinction of hepatitis C from other liver diseases is its propensity to cause extrahepatic manifestations, including joint pain (arthralgias), cryoglobulinemia, and various metabolic changes. This broad spectrum of potential effects highlights the complexity of hepatitis C, affecting not just liver function but also other bodily systems and requiring comprehensive management strategies.

## 3. Biology of Hepatitis C Virus and Its Association with Lipoproteins

### Characteristics of the Hepatitis C Virus

HCV is a particle between 50 and 80 nm in diameter containing a single-stranded RNA genome, nucleus, E1 and E2 glycoproteins, and type I transmembrane proteins, which form covalent bonds with infected hepatocytes [7]. They are closely associated with lipoproteins, which gives them a very low density [8]. The interactions governing the relationship between HCV virions and the different lipoproteins involved remain to be fully characterized [9].

**Figure 1:**
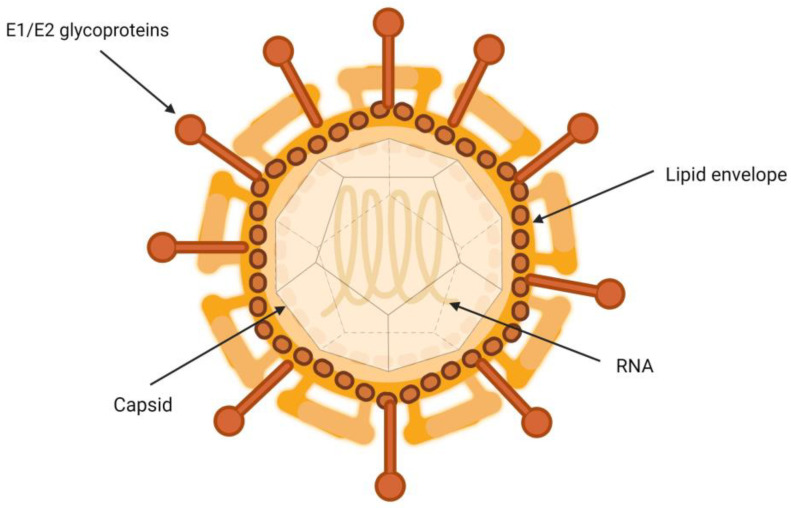
Structure of hepatitis C virion.

It has been suggested that HCV virion is a hybrid, consisting of a viral part merged with a lipoprotein capsule (Figure 1) [10]. Another hypothesis is that the relationship occurs through the interaction of apolipoproteins and lipid molecules that are part of the HCV envelope [11]. In both cases, the interaction with host lipoproteins could contribute to protecting and concealing the virion particles, covering their surface. This glycoprotein coat is essential in the process of inclusion of the viral particle into the target cells. It plays a crucial role in the binding and fusion process between the viral envelope and the endosomal membrane of the host cells [12].

In viral replication, HCV relies on the host cellular mechanism, which is associated with endoplasmic reticulum-derived membranes and various proteins [13]. HCV induces a massive reorganization of intracellular membranes, creating a membranous network [14].

Several electron microscopic studies have shown that the predominant structure is a double membrane vesicle consisting of proteins and cholesterol, as well as deposits of triglycerides (TGs) and cholesterol esters [15,16,17]. HCV alters the expression of genes involved in lipid metabolism, resulting in the accumulation of intracellular lipids [18].

## 4. Lipoproteins

Lipoproteins serve as vehicles for lipid transport, consisting of a nonpolar core filled with triglycerides (TGs) and esterified cholesterol, encased in a polar outer layer composed of apoproteins, phospholipids, and free cholesterol. This diverse group includes chylomicrons, very low-density lipoproteins (VLDLs), intermediate-density lipoproteins (IDLs), low-density lipoproteins (LDLs), and high-density lipoproteins (HDLs). The diversity among these lipoproteins lies in their free cholesterol and TG content and their unique compositions of apolipoproteins, reflecting their varied roles in lipid transport and metabolism within the body [19]. This variation emphasizes the complexity of lipid dynamics and their critical functions in maintaining cellular and systemic health.

Lipoprotein metabolism encompasses both exogenous and endogenous pathways. The exogenous pathway involves the absorption of dietary lipids through intestinal enterocytes, which are packaged into chylomicrons and enter the lymphatic system before reaching the bloodstream. On the other hand, the endogenous pathway occurs primarily in the liver (hepatocytes), where lipoproteins such as VLDL are synthesized and released into the circulation. These pathways are crucial for distributing lipids across different tissues for energy use, storage, or membrane synthesis.

### 4.1. Exogenous Pathway of Lipoprotein Metabolism

The lipids we obtain from the diet are mainly TGs. Once in the intestine, they bind to apoprotein B-48 in enterocytes, forming chylomicrons. These are secreted into the lymphatic vessels, reaching the general circulation via the thoracic duct. Chylomicrons become mature once they receive APOCII and APOE from HDL particles. There is also an exchange of TG with LDL particles located in the vascular endothelium, becoming remnant chylomicrons, taken up by hepatocytes through an interaction with APOE [20].

### 4.2. Endogenous Pathway of Lipoprotein Metabolism

The liver is the main organ involved in the endogenous lipoprotein metabolism pathway. Hepatocytes secrete VLDL, the formation of which is initiated in the sarcoplasmic reticulum by the incorporation of TG into APOB100 particles through the action of microsomal TG transfer protein. Cholesterol esters and APOE are incorporated into this particle. This is followed by the exocytosis of VLDL lipoproteins into the bloodstream, acquiring more APOE and APOC from the HDL particles [21]. Mature VLDLs are catabolized by the APOCII-activated enzyme lipoprotein lipase and renamed remnant VLDL or IDL. They are incorporated back into the liver through the interaction of APOE [22]. Alternatively, they are again hydrolyzed by hepatic lipase, whereby IDLs are transformed into LDLs, depleted of TGs and high in cholesterol. These particles transport cholesterol to peripheral tissues or the liver via APOB100 interactions with LDL receptors [23].

On the other hand, APOAI is the primary apolipoprotein of HDL particles [24]. It is again synthesized in the liver and intestine and is involved in forming these molecules through the esterification of cholesterol and phospholipids. During this process, the HDL molecules progressively lose part of their cholesterol load until they return to the hepatocyte or enterocyte, where they replenish their cholesterol stores [25].

## 5. Lipoprotein Profile Assessment

Dyslipidemia is a quantitative or qualitative alteration in circulating lipoproteins in plasma, notably an increase in the concentration of low-density lipoprotein cholesterol (LDL-cholesterol) [26]. However, episodes of atherothrombotic pathology are still observed in patients with normal or low cholesterol levels and without other known cardiovascular risk factors [27]. This suggests that there are other lipid alterations, beyond LDL cholesterol levels, that also increase cardiovascular risk [28]. The atherogenic potential of lipoproteins should be hence defined not just by their quantity but by their characteristics, including their number, size, and composition. Therefore, analyzing these aspects of lipoproteins provides a more comprehensive assessment of a patient’s lipid profile, offering insights beyond traditional cholesterol measurements [29,30].

As previously explained, lipoprotein particles differ from each other in terms of their free cholesterol and TG content. The relationship between density and size is inverse, with the smallest particles having the highest density [31].

These differences in the composition of the same class of particles influence the atherosclerotic process. Healthy vascular endothelium can be freely traversed by particles with diameters of less than 70 nm. These particles, especially smaller LDL particles, can be retained and are the origin of the atherogenic process [32].

It has also been observed that HDL particles can undergo modifications that change their structure and composition, thereby altering their function. For example, in diseases such as type II diabetes (T2DM), chronic kidney disease, sarcoidosis, and inflammatory processes, HDL particles lose their protective function and acquire an atherogenic effect [33].

The size of LDL lipoproteins is variable and depends on their core’s lipid content, which determines the particles’ density. This variability, which can be influenced by various alterations in lipoprotein metabolism, can lead to a discrepancy between the serum LDL cholesterol concentration and the number of circulating LDL particles [34]. Thus, many LDL particles may be associated with a normal LDL cholesterol concentration. This situation is known as c-LDL/p-LDL mismatch.

In these cases, different studies have found that particle number measurement is a better indicator than LDL concentration for assessing cardiovascular risk [35]. The prognostic ability of LDL particle number has been evaluated in different studies. For example, in the Framingham cohort, it was shown that an LDL particle concentration below the 25th percentile was a more reliable predictor of cardiovascular risk than an equivalent serum LDL cholesterol concentration [36]. Studies have even shown that treatment based on LDL particle number targets improves clinical outcomes over that based on LDL cholesterol concentration [37,38].

## 6. Alterations in Lipid Metabolism Associated with HCV Infection

**Figure 2:**
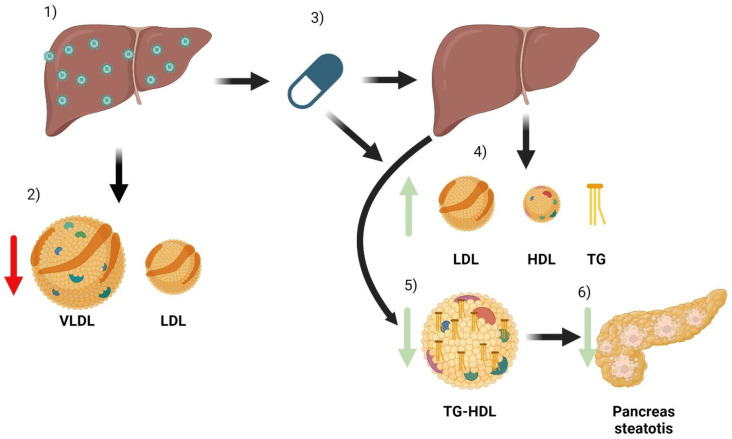
Summary of the main effects of HCV and its treatment on a patient’s lipid metabolism. When a patient has HCV infection, the main organ affected is the liver (1). Infection causes some changes in lipid metabolism, especially a decrease in the number of VLDL and LDL particles (2). After treatment with DAA (3), the infection is cured. Due to the combination of liver healing and the direct effect of antivirals, there are changes in lipoparticles. There is an increase in serum LDL, HDL, and triglyceride particles (4). In addition, improved liver function reduces the triglyceride content of HDL particles (5). HDL particles can now better mobilize lipids from tissues, which can reduce pancreatic steatosis and thus improve insulin resistance (6).

The most important complications associated with chronic HCV infection are liver cirrhosis and liver cancer. However, there are many extrahepatic manifestations that cause high morbidity and mortality [39]. Most are immunological or lymphoproliferative in origin, but alterations in the lipid profile have also been identified, leading to metabolic and cardiovascular complications [40]. The lipid profile’s modifications related to HCV infection and treatment are shown in Figure 2.

It is very striking that some studies have even been able to link the development of hepatocarcinoma with an alteration in oncogenesis. Moreover, this phenomenon is much more marked in HCV-infected patients than in HBV-infected patients. Some of the mediators involved could be AKT2, SREBP1c, and PPARγ. Also, some regulatory enzymes such as ACC and FAS may be involved [41].

Chronic HCV infection results in low levels of VLDL and LDL. Despite this apparently beneficial change, these patients have an increased development of atherosclerosis, leading to an increased cardiovascular risk [42]. This occurs independently of other risk factors, such as the development of T2DM or the presence of hepatic steatosis. Interestingly, HCV eradication leads to an increase in serum cholesterol and LDL levels, creating a combination of circumstances that may exacerbate the risk of atherosclerosis injury [43].

Another finding observed in patients with chronic HCV infection is the existence of abnormal lipoproteins, including VLDL particles enriched with TG, which increase atherogenic risk. These particles disappear after successful HCV treatment and cure.

However, it appears that the extent of this interaction is related to certain host polymorphisms and hepatitis C virus genotypes. Both factors are highly variable [44,45]. Evidence highlights that genotype 3 of the hepatitis C virus, accounting for 20–30% of infections, is particularly associated with the development of hepatic steatosis, exhibiting a more pronounced degree of steatosis in patients, even those without obesity, compared to other genotypes. This association extends to a direct correlation between viral load and steatosis severity, exclusively in genotype 3, a phenomenon not observed in other genotypes. Moreover, genotype 3 is linked to several adverse disease progression outcomes, such as increased treatment resistance and a higher risk of developing HCC [46].

The underlying mechanisms, though not fully understood, suggest that genotype 3 impacts key metabolic pathways involving microsomal triglyceride transfer protein (MTTP), sterol regulatory element-binding protein 1c (SREBP-1c), and peroxisome proliferator-activated receptor alpha (PPAR-α) [47]. This insight emphasizes the need for a genotype-specific approach in managing HCV infections, considering the unique challenges posed by genotype 3.

### 6.1. Diabetes Mellitus and Insulin Resistance

The development of T2DM is one of the most common HCV-related complications [48]. This relationship stems from a complex interplay between insulin resistance, hepatic steatosis, and inflammatory processes [49]. HCV-core transcription leads to an increased expression of TNF-alpha and thus to the induction of insulin resistance. This explains why the prevalence of T2DM is higher in patients with HCV liver disease compared to other etiologies of liver disease [50].

The development of T2DM can occur at any stage of liver disease, even with low degrees of fibrosis [51]. However, it is more prevalent in patients with advanced fibrosis or even liver cirrhosis [52]. As previously described, a genotype-dependent factor must be considered. Patients with genotype 3 have a higher risk of developing insulin resistance and diabetes. On the other hand, patients with genotype 1 would be more likely to improve their carbohydrate metabolism after a viral cure compared to genotypes 2 and 3 [53].

The development of T2DM correlates directly with the severity of liver fibrosis. Although it can occur in patients with mild fibrosis, the highest incidence is observed in those with liver cirrhosis. In addition, patients with HCV-associated T2DM have an increased risk of developing HCC. Regarding the relationship between T2DM and HCV treatment, early interferon treatment showed a worse response in patients with T2DM and HCV [54]. A decrease in the risk of de novo T2DM has been observed in several studies with the newer treatments, direct-acting analogues (DAAs) [55,56].

DAAs prevent the future onset of T2DM and improve glucose metabolism in patients who achieve sustained viral response (SVR). During follow-up, a decrease in glycated hemoglobin and an improvement in insulin resistance-related parameters have been observed. However, their long-term duration after achieving SVR needs to be better established [57].

### 6.2. Cardiovascular Diseases

HCV infection confers increased cardiovascular morbidity and mortality [58]. Early studies showed a relationship between HCV seropositivity and reduced carotid artery intima/media ratio. Subsequently, HCV was also found to cause an increased expression of pro-atherogenic cytokines [59,60].

Cardiovascular involvement appears to predominate in HCV patients compared to patients with other similar conditions, such as hepatitis B virus (HBV) [61]. This indicates that the cardiovascular risk is not solely due to liver damage but is an inherent effect of HCV itself.

In studies with large cohorts of patients with very long follow-up periods, it became evident that those patients who received antiviral treatment and achieved HCV eradication had lower mortality rates than those patients who did not receive treatment, not only due to hepatic but also extrahepatic causes, especially cardiovascular. Other studies showed improved myocardial perfusion in those patients who had SVR [62].

Another aspect to consider in the relationship between cardiovascular disease and hepatitis C is the interaction between their respective treatments. Antihypertensive drugs and statins are among the most frequently used simultaneously in patients receiving direct-acting antivirals. About 10% of patients took a statin before starting antiviral treatment [63]. Therefore, it is particularly important to consider interactions between these drugs [64]. Not all statins interact in the same way with all antivirals, although the most common complication is the development of myopathies and the need to lower the dose. Specific combinations, such as glecaprevir/pibrentasvir with atorvastatin, lovastatin, or simvastatin, as well as ledipasvir/sofosbuvir with rosuvastatin are formally contraindicated [65].

## 7. Metabolic Changes Related to Treatment with Direct-Acting Antivirals

Treatment with DAAs leads to changes in lipid metabolism. Serum total cholesterol and LDL-cholesterol levels rise, possibly increasing the risk of atherosclerotic lesions [66,67].

However, the results of studies assessing this point are sometimes contradictory. Some show increased HDL-cholesterol levels that are not seen in other studies. One study even described a decrease in HDL levels [68]. Regarding changes in TG, inconsistent results have also been reported: decreases, minimal or absent changes, or even increases in serum levels. The main clinical studies can be found summarized in Table 1.

These contradictory results may be explained by the heterogeneity of the studies, which were conducted in disparate genotypic populations, using different treatment regimens and different proportions of patients with liver cirrhosis [69,70]. Some studies included HIV-co-infected patients with an HIV-positive population of up to 60%. Follow-up times also varied widely between studies, ranging from 4 to 48 weeks. In addition, only two studies presented long-term prospective follow-up, Shimizu et al. and Gonzalez-Colominas et al. However, these two studies presented populations with liver involvement that goes beyond simple HCV infection: 50% of patients in the Gonzalez-Colominas study had liver cirrhosis and all patients in the Shimizu study had hepatic steatosis [71,72].

Critical questions about lipid changes post-DAA treatment include whether the initial changes persist over time and the effects on patients with early-stage liver disease, a group significantly understudied.

More recently, and especially after the generalization of DAAs drugs, lipid profile alterations have been described after HCV eradication [70]. In a study tracking HCV patients treated with DAAs for two years, total cholesterol and LDL cholesterol levels rose progressively, by an average of 15% and 22%, respectively. This led to a higher risk of cardiovascular events. An increase in LDL-C of more than 40% emerged as the sole predictive factor, suggesting it could be a warning sign for potential cardiovascular events in the HCV-eradicated population [73,74,75].

Total cholesterol and LDL-C increased earlier after DAA initiation, while TG and HDL-C increased slowly after the end of therapy [76]. This is consistent with the finding that rapidly elevated total cholesterol and LDL-C levels may correlate with rapid viral clearance due to potent DAA therapy. In addition, elevated lipid levels were not transient, but persisted years after the end of treatment. Age and smoking were factors associated with pronounced lipid changes after viral eradication. Patients with a history of untreated dyslipidemia had elevated lipid levels in the post-RVS state. All the above factors were also risk factors for cardiovascular/cerebrovascular events [77].

However, increases in HDL and TG levels remain controversial, with no apparent relationship established. Studies comparing the change in cholesterol levels before and shortly after DAA treatment and studies with a long follow-up period are scarce.

In recent studies, it has been discovered that the analysis of lipoparticle metabolism is more complex than initially thought and that not only the quantity but also the quality of lipoparticles determines the cardiovascular risk of patients [77]. Our results describe how HCV-dependent lipid abnormalities are associated with insulin resistance and how DAA therapy can reverse this association. These findings suggest that monitoring the HDL-TG profile could predict changes in glucose tolerance and insulin resistance post-HCV clearance [78].

**Table 1:** Principal clinical studies evaluating modifications in lipid profile after HCV treatment.

Study	Year	N	Design	Follow-Up	Drug	Genotype	Cirrhosis	Total Cholesterol	HDL	LDL	TG	Other
Chida T [79]	2018	70	Retrospective	4 weeks	DCL + ASV	1b	NA	↑↑	↑	↑↑	NA	
Endo [80]	2014–2016	276	Retrospective	24 weeks	DCV + ASV SOF + LDV SOF/LDV	1b	NA	↑↑	↑	↑↑	NA	
Inoue [67]	2018	216	Prospective post Hoc	48 weeks	DCV + ASV SOF/RBV	1b y 2	NA	↑↑	↑	=	=	
Meissner [66]	2015	54	Retrospective	24 weeks	SOF/RBV	1	NA	NA	NA	↑↑	↓	
Chaudhury [81]	2011–2017	251	Prospective Ad hoc	28 months	NA	1	NA	NA	NA	↑↑	↓	30% HIV
Sun [70]	2018	24	Prospective	ND	EBV/GPV LDV/SOF	1	NA	↑	NA	NA	↓	
Townsend [82]	2016	90	Prospective post Hoc	24 weeks	LDV/SOF SOF/LDV	1	NA	NA	NA	↑↑	NA	60% HIV
Morales [69]	2014–2016	52	Retrospective	6 months	SOF IFN LDV SIM/RBV	All	24%	↑↑	↓	↑↑	↓	
Beig [83]	1998–2016	132	Retrospective	48 weeks	DAA without IFN	All	No	↑↑	NA	↑↑	NA	Transplanted
Carvalho [84]	2018	178	Prospective	ND	DAA without IFN or RBV	All	NA	↑↑	NA	↑↑	↓	
Gitto [76]	2015	100	Prospective	24 weeks	DAA + RBV	All	80%	↑↑	NA	NA	NA	
Mauss [68]	2017	520	Prospective	ND	DAA	All	NA	↑↑	NA	↑↑	=	
El Sagheer [85]	2018	80	Retrospective	ND	SIM/SOF	4	>50%	↑↑	↑	↑↑	↓	
Gonzalez Colominas [86]	2019	226	Prospective	48 weeks	DAA	All	50%	↑↑	↑	↑↑	NA	
Doyle [87]	2015–2016	24	Prospective	24 weeks	OBV/DSV	All	NA	↑↑	↑	NA	↑	Evaluation of APOA, APOB and APOE, HOMA-IR
Ichikawa [88]	2014–2016	39	Prospective	24 weeks	DCV/ASV	1b	NA	↑↑	=	↑↑	=	
Shimizu [89]	2012–2016	70	Retrospective	48 weeks	All	1 y 2	NA	=	↑	↑↑	=	All patients with steatosis
Cheng [90]	2017	102	Prospective	12 weeks	All	All (1b 80%)	75%	↑↑	=	↑↑	↑	
Jain [91]	2017	50	Prospective	12 weeks	SOF/DCV	NC	NA	↑↑	=	↑↑	=	
Petta [92]	2018	182	Prospective	48 weeks	All	All	66%	↑↑	NA	NA	NA	
Casas-Deza [78]	2019	177	Prospective	48 weeks	All	All	10%	↑↑	↑	↑↑	↑	

ASV: Asunaprevir; DAA: direct acting antiviral; DCV: Daclatasvir; DSV: Dasabuvir; EBV: Elbasvir; GPV: Grazoprevir; IFN: Interferon; LDV: Ledipasvir; NA: not available; ND: No data; OBV: Ombitasvir; RBV: Ribavirin; SOF: Sofosbuvir; SIM: Simeprevir; TG: triglycerides; ↑: slow increase; ↑↑: rapid increase; ↓: slow decrease; =: no changes.

Further, it has been found that post-treatment, the TG content in HDL particles decreases, signifying improved lipoparticle quality and enhanced cholesterol clearance from tissues. This reduction in hepatic and pancreatic fat could partly account for the observed improvement in insulin resistance [93].

Various LDL-C reduction thresholds (70, 100, and 155 to 190 mg/dL) are recommended to lower the risk of atherosclerotic cardiovascular disease (ACVD) [94]. These recommendations consider factors like initial LDL-C levels, age, ethnicity, and the estimated future risk of cardiovascular disease. Current evidence regarding the management of patients with dyslipidemia seems to favor the “lower is better” concept [75]. Due to lower lipid profiles prior to anti-HCV therapy, deteriorating lipid profiles are often overlooked in the post-HCVR era; 7.3% of patients without concurrent lipid-lowering therapy prior to antivirals were started on lipid-lowering drugs during the follow-up period. In this study, after excluding patients who took lipid-lowering drugs both before and after anti-HCV therapy, the proportion of patients with LDL > 100 mg/dL increased from 37.5% before treatment to 56.9% after anti-HCV therapy, while the proportion of patients with LDL levels >155 mg/dL increased from 2% before treatment to 7.2% after antiviral therapy [95].

A significantly higher proportion of patients justified the use of lipid-lowering drugs to reduce the risk of vascular events in the post-antiviral treatment era. However, it is believed that lipid-lowering therapy may be significantly underutilized in this population [95].

It has been suggested that HCV eradication reduces the risk of cerebrovascular events. In a recent study, 731 of 17,103 treated patients who achieved SVR experienced cardiovascular events during the follow-up period (19.1 per 1000 person-years) [76,96]. A 13% risk reduction was observed in patients with coronary heart disease receiving interferon- or AAD-based regimens compared to the untreated cohort. However, another large cohort study of 160,875 subjects revealed that the benefit of HCV eradication was only found to reduce the risk of stroke, but not coronary heart disease, compared to the untreated cohort [78,97].

Notably, most of the patients who developed cardiovascular disease after HCV treatment had no obvious risk factors prior to antiviral therapy [23,98].

Other studies have shown that DAAs improve carotid thickening, but carotid plaques did not change in the same cohort [94,99]. Meanwhile, a recent study has shown dyslipidemia and a short-term increase in aortic stiffness in patients with advanced fibrosis after DAA treatment. Overall, the improvement in vascular events in the post-SVR state must be judged on an individual basis, considering lipid dynamics.

However, most existing studies after the advent of DAAs have a relatively short follow-up period, and there are no HCV-uninfected controls. In addition, the number of patients with vascular events is also limited, making it difficult to draw conclusions. This situation underscores the necessity for future studies to investigate whether the potential increase in LDL cholesterol levels following DAA treatment could be counterbalanced by a decrease in systemic inflammation among patients who achieve a SVR. Such research is paramount for clarifying the risk of developing cardio-cerebrovascular diseases in this patient population.

In conclusion, after achieving SVR through DAA treatment, monitoring for both hepatic and extrahepatic outcomes is crucial, given the known lipid changes and their potential impact on cardiovascular health. While current guidelines suggest discharging patients without advanced liver disease post-SVR [95,100], the observed increases in total and LDL cholesterol post-treatment highlight the need for ongoing vigilance against vascular events and cardio-cerebrovascular diseases. Recent findings of improved lipoprotein quality and decreased TGs post-HCV cure, potentially reducing insulin resistance and cardiovascular risk, underline the importance of extended follow-up and larger studies to understand these long-term effects fully.
